# Reliability and Validity of Ecological Momentary Assessment Response Time–Based Measures of Emotional Clarity: Secondary Data Analysis

**DOI:** 10.2196/58352

**Published:** 2024-07-18

**Authors:** Raymond Hernandez, Claire Hoogendoorn, Jeffrey S Gonzalez, Elizabeth A Pyatak, Gladys Crespo-Ramos, Stefan Schneider

**Affiliations:** 1 Center for Economic and Social Research University of Southern California Los Angeles, CA United States; 2 Chan Division of Occupational Science and Occupational Therapy University of Southern California Los Angeles, CA United States; 3 Ferkauf Graduate School of Psychology Yeshiva University Bronx, NY United States; 4 Department of Medicine (Endocrinology) Albert Einstein College of Medicine Bronx, NY United States; 5 Department of Psychology University of Southern California Los Angeles, CA United States

**Keywords:** digital mental health, drift-diffusion model, ecological momentary assessment, emotional clarity, emotional health, emotion regulation, response time, positive affect, negative affect, type 1 diabetes, mobile phone

## Abstract

**Background:**

Emotional clarity has often been assessed with self-report measures, but efforts have also been made to measure it passively, which has advantages such as avoiding potential inaccuracy in responses stemming from social desirability bias or poor insight into emotional clarity. Response times (RTs) to emotion items administered in ecological momentary assessments (EMAs) may be an indirect indicator of emotional clarity. Another proposed indicator is the *drift rate* parameter, which assumes that, aside from how fast a person responds to emotion items, the measurement of emotional clarity also requires the consideration of how careful participants were in providing responses.

**Objective:**

This paper aims to examine the reliability and validity of RTs and drift rate parameters from EMA emotion items as indicators of individual differences in emotional clarity.

**Methods:**

Secondary data analysis was conducted on data from 196 adults with type 1 diabetes who completed a 2-week EMA study involving the completion of 5 to 6 surveys daily. If lower RTs and higher drift rates (from EMA emotion items) were indicators of emotional clarity, we hypothesized that greater levels (ie, higher clarity) should be associated with greater life satisfaction; lower levels of neuroticism, depression, anxiety, and diabetes distress; and fewer difficulties with emotion regulation. Because prior literature suggested emotional clarity could be valence specific, EMA items for negative affect (NA) and positive affect were examined separately.

**Results:**

Reliability of the proposed indicators of emotional clarity was acceptable with a small number of EMA prompts (ie, 4 to 7 prompts in total or 1 to 2 days of EMA surveys). Consistent with expectations, the average drift rate of NA items across multiple EMAs had expected associations with other measures, such as correlations of *r*=–0.27 (*P*<.001) with depression symptoms, *r*=–0.27 (*P*=.001) with anxiety symptoms, *r*=–0.15 (*P*=.03) with emotion regulation difficulties, and *r*=0.63 (*P*<.001) with RTs to NA items. People with a higher NA drift rate responded faster to NA emotion items, had greater subjective well-being (eg, fewer depression symptoms), and had fewer difficulties with overall emotion regulation, which are all aligned with the expectation for an emotional clarity measure. Contrary to expectations, the validities of average RTs to NA items, the drift rate of positive affect items, and RTs to positive affect items were not strongly supported by our results.

**Conclusions:**

Study findings provided initial support for the validity of NA drift rate as an indicator of emotional clarity but not for that of other RT-based clarity measures. Evidence was preliminary because the sample size was not sufficient to detect small but potentially meaningful correlations, as the sample size of the diabetes EMA study was chosen for other more primary research questions. Further research on passive emotional clarity measures is needed.

## Introduction

### Background

Emotion regulation is highly relevant to subjective well-being from both hedonic and eudaimonic perspectives. According to the hedonic perspective, well-being is the experience of happiness or the occurrence of positive affect (PA) and absence of negative affect (NA) [1]. In the eudaimonic perspective, well-being arises when individuals live with a sense of growth, meaningfulness, and purpose [2]. People often engage in emotion regulation to obtain hedonic benefits (eg, feel more PA and reduce NA) [3], but eudaimonic motivations have been found to be important as well [4] (eg, downregulation of negative emotions to achieve a sense of growth in one’s ability to handle daily stressors).

An important aspect of emotion regulation is emotional clarity, a person’s ability to lucidly identify the type of emotion they are experiencing [5]. Emotional clarity is highly relevant to the James Gross Model of Emotion Regulation [6]. The model consists of emotion regulation strategies that are either “antecedent-focused” or “response-focused,” which refers to whether the strategy is used before or after an emotional response fully develops. In the updated emotion regulation model of Gross, understanding and identifying one’s emotions accurately (ie, emotional clarity) are precursors to both these types of emotion regulation strategies [7]. Therefore, individuals with low emotional clarity are less likely to use emotion regulation strategies (as they are failing to identify the need for them), which can negatively impact well-being. Lower emotional clarity has often been associated with reduced mental health [8-10], although there are exceptions. For instance, prior research has suggested that higher emotional clarity may be adaptive primarily for individuals who do not have very frequent and strong experiences of negative emotions but maladaptive for those who frequently have strong feelings of NA [11].

Both direct and indirect measures of emotional clarity have been developed [12,13]. Direct assessments involve the metacognitive task of reflecting on one’s emotional clarity level, while indirect assessments measure the performance of a task relevant to emotional clarity (ie, answering emotion items) and do not require self-insight [12]. Emotional clarity is commonly directly assessed with cross-sectional self-report measures such as the clarity subscales of the Trait Meta-Mood Scale [14] and the Difficulties in Emotion Regulation Scale (DERS) [15], but indirect measures have been argued to have potential advantages over self-report assessments [12,13]. For instance, they could help avoid possible issues with subjective reports, including poor insight into emotional clarity and the possibility of social desirability bias (ie, participants not wanting to report uncertainty about their feelings) [12,16]. However, for the potential utility to be realized, further investigations of the validity of indirect measures of emotional clarity are needed.

### Assessing Emotional Clarity With Item Response Times

Response times (RTs) to emotion questions in ecological momentary assessments (EMAs) have been argued to be indirect measures of emotional clarity at the momentary (within-person) level [12]. Theoretically, the greater an individual’s momentary affective clarity, the less time should be needed to provide a rating of momentary affect [12], whereas longer RTs to affect items should be indicative of lower emotional clarity. Evidence supporting this theory has been found, such as shorter RTs to affect items being associated with better momentary emotion regulation and mood [12]. However, emotional clarity may be confounded with emotional intensity [9], and evidence suggests the validity of RTs as a measure of affective clarity is enhanced by controlling for emotional intensity at the within-person (and not between-person) level [17].

There was no strong evidence that the study-long aggregates of emotion item RTs (between-person level) could act as indicators of trait emotional clarity [12,17]. These aggregates were found to not have significant relationships with global measures of emotional clarity and have inconsistent relationships with global emotion regulation measures [12,17]. Suggested reasons for the lack of a relationship between global measures of emotional clarity and the aggregate of emotion item RTs include a modality difference (ie, self-report vs indirect behavior-based assessment), low conceptual correspondence (ie, different forms of emotional clarity are being assessed), and difference in assessment timing (ie, 1 time vs repeated EMA measurements) [12,17]. The relationships between the study-long aggregate of emotion item RTs and subjective well-being variables of relevance to emotional clarity (eg, depression and anxiety) [8,18] were not examined, which could have served as useful additional convergent validity tests. Finally, the possibility that processing speed had a confounding effect on correlations between study-long aggregates of emotion item RTs and other variables was not investigated. In prior research, RTs to emotion EMA items have been found to have moderate correlations with processing speed measures [19], suggesting that individual differences in emotion item RTs may at least in part be attributable to processing speed.

### Assessing Emotional Clarity With the Drift Rate Parameter

Another indirect measure of emotional clarity was recently proposed, drift rate, which is computed using the drift-diffusion model [20]. This model, which is often used in cognitive psychology, was explicitly developed to disentangle different components of RTs [21]. The drift-diffusion model proposes that decisions (eg, choosing responses on EMA items) are made through the accumulation of information until a threshold of sufficient information (as determined by the individual) is reached [21]. Decisions can be fast if the speed of information accumulation (ie, drift rate) is fast, if the threshold for a decision (ie, boundary separation or response caution) is low, or both. The D-diffusion item response theory (IRT) model is an IRT version of the drift-diffusion model that was specifically designed for the analysis of self-report ratings, such as emotion ratings [22]. In the context of answering mood questions, the D-diffusion IRT model additionally considers that RTs may also be fast if extremes of the emotion are experienced (ie, very high or low levels), making the provision of ratings more straightforward [20]. When the D-diffusion model is applied to EMA responses and their RTs, it can take into account all the aforementioned influences on RTs and output drift rate. In the EMA context, drift rate can be interpreted as the speed of access to information relevant to the question being asked [20,21], which here is information regarding emotions. A more detailed description of how the drift rate parameter was computed can be found in the *Emotional Clarity Measure 2: Drift Rate From the Drift-Diffusion Model* subsection in the *Methods* section.

A person would be considered to have high emotional clarity when responding both fast and carefully to emotion items, which differs from how emotional clarity assessed via RTs considers only speed. As the absolute difference between a person’s NA level and the level of NA captured by an item increases, the D-diffusion model assumes that an individual would be expected to have faster RTs as a result of the so-called “distance-difficulty” principle, a well-established phenomenon in the RT literature [23], whereby the more items contrast with a person’s state, the easier they are. For instance, an individual with a very low NA would be expected to quickly respond to an item asking about being scared (an item often associated with a high NA) [24], whereas the same individual would be expected to require a longer RT for an item asking about irritability (which has been associated with a low NA) [24]. According to the D-diffusion model, response caution is low when a person responds similarly quickly (or slowly) to all items regardless of their content (an indication that the person did not answer carefully). Assuming a particular observed item RT, when response caution is lower (ie, less information is gathered), drift rate (ie, the rate of information accumulation or information divided by time) would also be lower. Conversely, for a person with a higher response caution, the estimated drift rate (and emotional clarity) would be greater for the same RT.

Our initial evidence suggested that the drift rate parameter derived from EMA NA items may be an indicator of emotional clarity of low versus high NA, including expected relationships (ie, negative associations) at the between-person level with neuroticism and depression [20], but we did not account for factors aside from emotional clarity that could impact drift rate. For instance, drift rate could be impacted by individual differences in the general speed of responding to questionnaires (eg, due to reading speed, motor behavior, and familiarity with computer use). Studies on emotional clarity measured by survey item RTs had accounted for individual differences in this baseline speed of responding by adjusting for it [12], but this was not done in our prior study [20]. Cognitive processing speed was hypothesized to also potentially impact drift rate but was also not adjusted for, as the measure was not available in the prior study [20].

### Objectives

#### Trait Emotional Clarity

There has been substantial prior research and interest in examining emotional clarity as a trait [12,14,15], but further research on the indirect (instead of direct) measurement of individual differences in emotional clarity via EMA data is needed. Compared to traditional cross-sectional self-report measures, indirect measurement of individual differences in emotional clarity potentially has the advantage of less susceptibility to self-report biases stemming from causes such as social desirability and poor insight [12,16]. Furthermore, if an emotion is being assessed via EMA, *indirect assessment of emotional clarity via emotion items affords the possibility of capturing individual differences in emotional clarity without burdening participants with additional items*. The use of EMA methodology is ubiquitous in a broad range of fields [25-27], and the use of EMA emotion items is commonplace [28]. Indirect assessment of trait emotional clarity would, therefore, make the investigation of emotional clarity possible for a large number of EMA data sets without additional emotional clarity items.

The focus of this paper was to examine the reliability and validity of 2 RT-based indirect indicators of individual differences in emotional clarity among adults with type 1 diabetes (T1D). One indicator was the average of repeated measures of RTs to emotion items, and the other was the average of repeated measures of drift rate (ie, the speed of accessing information about one’s current affect) derived from emotion items. In validity testing, in contrast to prior work [20], *individual differences in the baseline speed of responding and processing speed were controlled for*. We examined data from an EMA study of adults with T1D [29]. EMA surveys (which included emotion items) were completed 5 to 6 times a day (depending on the participants’ sleep schedule) for 2 weeks, and the RTs for each item were recorded. Notably, RT-based metrics can vary within people, and their study-long averages can vary across people. Multilevel modeling was used to account for both these potential sources of variance.

Emotional clarity may be of particular relevance for adults with T1D. Compared to the general population, adults with T1D may be exposed to stressors more frequently, specifically emotional distress related to the daily care of diabetes. A prior study estimated that performing all recommended diabetes self-management activities would require >2 hours daily [30]. Given such burden and associated diabetes distress (emotional distress specific to the daily care of diabetes), individuals with diabetes are more likely to experience lower subjective well-being, such as more symptoms of depression, compared to healthy populations [31]. Diabetes distress has been found to be negatively associated with emotion regulation ability [32]. Therefore, emotional clarity, a possible precursor to emotion regulation [10], may be integral for adults with T1D to cope with the burden of the condition. Poor coping with diabetes can lead to the neglect of diabetes self-management behaviors, which can amplify the health consequences of diabetes. Greater diabetes distress has been associated with lower adherence to insulin regimens [33,34], while greater depressive symptoms have been associated with lower adherence to diet, exercise, and glucose testing recommendations [35].

#### Reliability

The reliability of the proposed measures for emotional clarity was investigated by examining the test-retest stability of the measures across EMA measurement occasions.

#### Validity

The validity of the RT-based indicators of emotional clarity was tested by examining their associations with well-validated measures of relevance to emotional clarity. In forming our hypotheses (summarized in Table 1), we made a distinction between the clarity of PA and the clarity of NA because of prior research suggesting that the latter had associations with mental health while the former did not [9]. Therefore, we speculated that the awareness of NA is a more direct precursor to the application of coping strategies and successful coping than the awareness of PA.

**Table 1:** Hypothesized associations of the negative affect (NA) and positive affect (PA) clarity indicators with subjective well-being and emotion regulation.

		Emotional clarity indicator			
		NA drift rate	PA drift rate	RT^a^ for NA items^b^	RT for PA items
**Subjective well-being**					
	Satisfaction with life	Positive	Null	Positive	Null
	Neuroticism	Negative	Null	Negative	Null
	Depression	Negative	Null	Negative	Null
	Anxiety	Negative	Null	Negative	Null
	Diabetes distress	Negative	Null	Negative	Null
**Emotion regulation and its 6 components**					
	Emotion regulation difficulties (overall)	Negative	Null	Negative	Null
	Limited strategies	Negative	Null	Negative	Null
	Nonacceptance	Negative	Null	Negative	Null
	Impulse control difficulties	Negative	Null	Negative	Null
	Difficulties with goal directedness	Negative	Null	Negative	Null
	Lack of awareness	Negative	Negative	Negative	Negative
	Lack of emotional clarity	Negative	Negative	Negative	Negative

^a^RT: response time.

^b^Multiplied by –1 so that higher values indicate greater emotional clarity.

#### Hypothesized Associations With Subjective Well-Being

Past literature has found that increased emotional clarity and emotion regulation ability, as assessed by questionnaires or RTs to emotion items, were associated with greater subjective well-being, including greater life satisfaction [36], lower neuroticism [9], lower depression [18], fewer anxiety symptoms [8], and less diabetes distress [32]. However, following the results of the study by Thompson et al [9], we hypothesized that these relationships should hold only for indicators of NA clarity, not PA clarity.

#### Hypothesized Associations With Emotion Regulation

We considered the valence of emotions in the generation of our hypotheses regarding associations between passively collected indicators of emotional clarity and emotion regulation, given previous work showing that the clarity of positive emotions had different associations with other measures (eg, neuroticism and depression) compared to the clarity of negative emotions [9]. The DERS [15] has 6 subscales representative of emotion regulation components (listed in Table 1), as well as an overall score. Of the 6 DERS subscales, 4 (67%) specifically assess problems with regulating NA (ie, limited access to emotion regulation strategies when upset, nonacceptance of negative emotional responses, impulse control difficulties when upset, and difficulties with goal-directed behaviors when upset); we hypothesized that greater difficulties indicated on each of these component scales would be associated with lower NA clarity on the proposed RT-based measures [10], whereas we did not expect them to be associated with PA clarity on the RT-based measures (Table 1). That is, we expected NA clarity to precede and hence be more relevant to NA regulation than PA clarity, consistent with prior findings of associations between neuroticism and NA clarity, but not PA clarity [9]. The remaining 2 (33%) components of emotion regulation (ie, a lack of emotional awareness and lack of emotional clarity) are not specific to the regulation of NA [15]; therefore, we hypothesized that they would be associated with the clarity of both NA and PA using the RT-based measures. Notably, 1 of these 2 components assesses self-reported emotional clarity; we expected that this component would have associations of a greater magnitude with RT-based measures of NA and PA clarity compared to other emotion regulation subscales. Finally, we hypothesized an association between the DERS total score and indicators of NA clarity but not indicators of PA clarity because 4 (67%) of the 6 DERS subscales were relevant to emotion regulation when experiencing NA.

#### Adjustment by Individual Differences in RT and Processing Speed

We examined whether adjusting the RT-based emotional clarity indicators by individual differences in RT and participant processing speed would affect the results of the convergent or divergent validity tests. Both RTs to survey items and drift rate were expected to not be purely indicators of emotional clarity but likely also be impacted by processing speed [19,20,37] and differences in the baseline speed of responding [12]. Therefore, we examined the robustness of the results of validity testing when statistically adjusting for both these variables.

## Methods

### Overview

The analyzed data were from an EMA study focused on investigating the relationships among momentary blood glucose level, emotional state, and functioning in adults with T1D [29]. Participants were recruited from 3 clinical sites through health care provider referrals, mailings, flyers, and emails. Inclusion criteria were having a diagnosis of T1D, being able to speak and read English or Spanish, and the ability and willingness to carry out study procedures (eg, completion of EMAs and cognitive tests on smartphones). Study procedures included the completion of baseline surveys, training in the use of study devices, 2 weeks of 5 to 6 EMAs and ambulatory cognitive tests daily, and follow-up surveys. EMA surveys began at participants’ selected wake-up time each day and were administered at 3-hour intervals after that until sleep time. If a participant reported that they would likely be sleeping by the time of the sixth survey (ie, 15 hours after the first survey), then they were given the option to complete 5 surveys daily instead of 6. To encourage EMA compliance, 3 brief check-in emails or calls were scheduled with study staff. In addition, if EMA survey completion was low, then study staff would contact the participant to query if any support was required. The study procedures are described in greater depth in a protocol paper [29].

### Ethical Considerations

All study procedures were approved by the University of Southern California’s Institutional Review Board (proposal #HS-18-01014). Informed consent was provided before study participation. Participants were compensated US $200 via a reloadable debit card for completion of all study procedures. Study data were deidentified before analysis.

### Measures

#### RT-Based Measures of Emotional Clarity

The clarity of PA was assessed with RTs to EMA items about how happy, content, enthusiastic, or excited participants felt “right now,” while the clarity of NA was assessed with RTs to EMA items asking how tense, upset, sad, or disappointed participants felt in the moment. These emotion adjectives were taken from the “Stress and Working Memory” study [38] and were chosen because they mapped neatly onto the circumplex model of affect [39]. That is, there were 2 items in each of the 4 circumplex dimensions (ie, unpleasant and activated, unpleasant and deactivated, pleasant and activated, and pleasant and deactivated), thereby ensuring that a range of emotion types was represented. The responses were all given on slider scales from 0 (not at all) to 100 (extremely). These emotion items were administered at every EMA prompt using the mobile EMA app (ilumivu [40]). Items were presented one at a time on study-provided smartphones. For each item, RTs were recorded in milliseconds. RTs that were deemed too fast (ie, <0.2 seconds) or too slow (ie, >30 seconds) were set to missing for analyses (5979/461,896, 1.29% of the observations) because such outliers could be indicative of careless responding or distractions during survey completion [20,41].

#### Emotional Clarity Measure 1: Median RTs

NA and PA clarity were computed as the median RT of the 4 NA items and the 4 PA items at each EMA prompt [12,17] (RTs were multiplied by –1 such that higher values indicate greater clarity). In this paper, median RT for NA items will be referred to as NA RT and median RT for PA items as PA RT.

Following prior research, the median RTs were adjusted for the baseline speed of responding to partial out individual differences in RT stemming from factors such as reading and screen tapping speed [12,17]. We assessed participants’ baseline speed by taking the median RT across all EMA occasions on a multiple-choice question asking what they were doing immediately before the survey.

#### Emotional Clarity Measure 2: Drift Rate From the Drift-Diffusion Model

Drift rates for both NA and PA items were calculated for each EMA following procedures described in detail in a prior study [20] with software code available [42]. In brief, we estimated the drift rates for each person and EMA measurement occasion using the IRT based-variant of the drift-diffusion model that was specifically developed for use with self-report (eg, EMA) items. Because the IRT model requires binary variables, responses to PA and NA items were converted into dichotomous variables such that responses below the midpoint of the scale were coded as 0, while responses at or above the midpoint of the scale were coded as 1. Previous analyses have shown that drift rate measures derived from continuous items that were dichotomized demonstrate convergent validity with items that were already presented to respondents in a binary response format [20]. Next, we examined whether the dichotomized PA and NA item sets were unidimensional, a condition necessary for the calculation of drift rate; we conducted a confirmatory factor analysis in Mplus (version 8.8; Muthén & Muthén) [43] using the weighted least square mean and variance adjusted estimator, using cluster-robust SEs to account for the nesting of multiple EMA measurement occasions within individuals. We examined whether fit indices were within the traditional ranges for acceptable model fit, including a root mean square error of approximation (RMSEA) of at least <0.08, comparative fit index (CFI) and Tucker-Lewis Index (TLI) of at least >0.90, and standardized root mean square residual (SRMR) of <0.08 [44]. Within and between-person reliability (McDonald omega) coefficients were also computed for the dichotomized and nondichotomized NA and PA items [45].

A drift-diffusion model was then applied to the RTs and dichotomized response values for PA and NA items using the *diffIRT* package in R (R Foundation for Statistical Computing) [22], where drift rate and RTs were modeled as latent factors. Factor score estimates for the drift rate parameter were then calculated for each EMA occasion, separately for NA and PA. To examine the fit of the drift-diffusion model on the PA and NA item sets, we examined the level of consistency between the observed and diffusion model–predicted RT distributions with histograms and density plots.

The drift rate parameters used in our primary analyses are a processed version of the drift rate parameters from the diffusion IRT models referred to as the *absolute drift rate*, computed as the mean absolute difference between the drift rate parameter factor scores and the item difficulty levels. The drift rate parameter is an estimation of a person’s tendency to report high NA (or PA) in a moment after taking into account both a person’s responses and item RTs. However, emotional clarity should be indicated by the speed in carefully accessing one’s mood regardless of its valence (eg, high or low NA). Therefore, following the distance-difficulty hypothesis-informed formula for the speed of information accumulation in the D-diffusion model [22], we found the absolute value of the difference between the drift rate parameter and average item difficulty and operationalized this absolute drift rate as emotional clarity. These absolute drift rates were then log transformed to normalize their distributions. When used in analyses, drift rates were also adjusted by baseline speed.

#### Other Measures

The measures used for convergent or divergent validity testing were completed before (“baseline”) or after (“follow-up”) the EMA study period. Life satisfaction was assessed with the Satisfaction with Life Scale [46], neuroticism with the Ten-Item Personality Inventory [47], depression with the Patient Health Questionnaire [48], anxiety with the Generalized Anxiety Disorder Scale [49], diabetes distress with the Problem Areas in Diabetes Scale short form [50], and emotion regulation with the DERS short form (DERS-SF) [15]. The Ten-Item Personality Inventory, Patient Health Questionnaire, Generalized Anxiety Disorder Scale, and DERS-SF were administered at baseline, while the Satisfaction with Life Scale and Problem Areas in Diabetes Scale short form were completed at follow-up [29].

Processing speed was assessed with the Symbol Search task [51], an ambulatory cognitive test administered as part of every EMA prompt [29]. The Symbol Search task captured perceptual speed, which is a component of processing speed [51,52]. Participants were presented with 2 cards at the top and 2 cards at the bottom of the phone screen, each with 2 symbols. As quickly as they could, they were asked to choose the card at the bottom of the screen that matched with one of the cards on top. The task consisted of 20 trials, and processing speed was measured as the median RT in accurate trials, only for sessions with at least 70% (14/20) matching accuracy [51]. Symbol Search RTs were calculated such that higher values indicate faster processing speed.

### Statistical Analyses

#### Reliability

Reliability was assessed for each of the emotional clarity indicators: NA drift rate, PA drift rate, NA RT, and PA RT. It was calculated using the following formula: between-person reliability=variance (between-person)/(variance [between-person] + variance [within-person]/n) [53], where between-person variance is the variance in the average of scores across measurement occasions (ie, EMA prompts), within-person variance is the variance of scores across measurement occasions within a person, and n is the number of measurement occasions. Between and within-person variance were calculated with multilevel models, with EMA prompts nested in individuals, where the measure of interest (eg, the NA drift rate) was specified at both levels 1 and 2. To examine how many measurement occasions would be needed to obtain acceptable reliability (≥0.7) for each emotional clarity indicator, we estimated how reliability changed as a function of the number of measurement occasions, moving from 2 EMA prompts to a maximum of 70 prompts. Mplus (version 8.10) [43] was used for reliability analyses via the package *MplusAutomation* [54] in the statistical software R (The R Foundation) [55].

#### Validity

Validity testing was performed for average NA drift rate, PA drift rate, NA RT, and PA RT. To account for the nested data structure, with multiple EMA prompts nested in individuals, we estimated correlation coefficients between the emotional clarity indicators and other measures using multilevel structural equation models (MSEMs). Multilevel variables with both within- and between-person variance (NA drift rate, PA drift rate, NA RT, PA RT, and processing speed) were specified at both level 1 (within-person) and level 2 (between-person), with latent means estimated at level 2 of the MSEM. As all cross-sectional measures only contained between-person variance, they were entered into the MSEM at level 2 and allowed to correlate with the NA drift rate, PA drift rate, NA RT, and PA RT variables. To adjust for individual differences in baseline speed, NA drift rate, PA drift rate, NA RT, and PA RT were regressed on baseline speed at level 2 of the MSEM. In addition, covariances were specified between baseline speed and all other level-2 variables. Prior research indicated that RTs to tasks were affected by the time of day [19,56]. Therefore, at level 1, all the RT-based metrics were adjusted for (ie, regressed on) the time of day, coded as a categorical variable, where a participant’s first survey per day was categorized as taking place in the morning, their final scheduled survey of the day as taking place in the evening, and all their surveys in between as taking place midday. The reference group was “midday,” meaning that at level 2, the latent means for the RT-based metrics were for midday surveys.

Various sensitivity tests were also conducted. Separate multilevel regression models explored whether adjustment for emotional intensity impacted the relationships between RT and each cross-sectional measure, such that NA RT and PA RT were additionally regressed on linear and quadratic terms of overall NA and PA ratings (ie, average rating across NA or PA items within an EMA survey), respectively. In supplemental analyses in which all the proposed emotional clarity indicators were controlled for processing speed, they were all regressed on processing speed at level 2. Finally, we tested whether the association between NA and PA drift rate and other measures would differ if the drift metrics were computed from mood items that were dichotomized at each person’s mean NA and PA response rather than the scale midpoint. For all analyses, data from all participants were included regardless of completion rates because MSEMs estimate latent averages of variables at level 1 that account for potential unreliability stemming from sparse participant data [57]. All validity analyses were conducted in M*plus* (version 8.10) [43] using maximum likelihood with robust SEs. Code for both the reliability and validity analyses is provided [58].

## Results

### Descriptive Statistics

Characteristics of the study sample, 196 adults with T1D, are shown in Table 2. The median EMA completion rate over the 2-week study period was 92% (IQR 11%).

Descriptive statistics for the EMA variables are presented in Table 3. Distributions for RTs to individual NA and PA items are shown in Figures S1 and S2 in Multimedia Appendix 1, respectively. Tables S1 and S2 in Multimedia Appendix 1 show the between-person correlations between (unadjusted) study measures.

A unidimensional model was found to fit both the (dichotomized) 4 NA and 4 PA items acceptably, justifying the calculation of drift rates for both types of items. For NA, *χ*^2^_2_=10.6, *P*=.005; CFI=0.998; TLI=0.994; RMSEA=0.018; and SRMR=0.018, while for PA, *χ*^2^_2_=53.3, *P*<.001; CFI=0.989; TLI=0.966; RMSEA=0.044; and SRMR=0.031. All these values were within commonly suggested ranges for acceptable model fit [44]. The within-person omega estimate for the 4 dichotomized NA items was 0.710, while the between-person omega estimate was 0.938. For the 4 dichotomized PA items, the within-person omega estimate was 0.674, and the between-person omega estimate was 0.939. The within-person omega estimate for the 4 nondichotomized NA items was 0.793, while the between-person omega estimate was 0.955. For the 4 nondichotomized PA items, the within-person omega estimate was 0.773, and the between-person omega estimate was 0.937. For all PA and NA items, the observed RT distributions (histograms) were consistent with the drift-diffusion model–predicted RT distributions as per density plots of these RTs (Figures S1 and S2 in Multimedia Appendix 1), indicating a good fit overall for the D-diffusion IRT model [22].

**Table 2:** Participant characteristics (N=196).

Characteristic			Values
Age (y), mean (SD; range)			39.6 (14.3; 18-75)
**Sex n (%)**			
	Male	88 (44.9)	
	Female	108 (55.1)	
	Other	0 (0)	
**Ethnicity, n (%)**			
	African American	29 (14.8)	
	Asian	7 (3.6)	
	Latino	80 (40.8)	
	White	56 (28.6)	
	Multiethnic	14 (7.1)	
	Other	6 (3.1)	
	Not reported	4 (2)	
**Employment status, n (%)**			
	Full time	69 (35.2)	
	Part time	23 (11.7)	
	Full-time homemaker	9 (4.6)	
	Student	18 (9.2)	
	Unemployed	27 (13.8)	
	Retired	15 (7.7)	
	Disabled	23 (11.7)	
	Other	8 (4.1)	
	Not reported	4 (2)	
**Annual household income (US $), n (%)**			
	<25,000	47 (24)	
	25,000-49,999	43 (21.9)	
	50,000-74,999	15 (7.7)	
	≥75,000	40 (20.4)	
	Not provided	51 (26)	
**Mental health^a^**			
	SWLS^b^ score, mean (SD; range)	22.0 (7.5; 5-35)	
	TIPI^c^ neuroticism score, mean (SD; range)	3.2 (1.3; 1-7)	
	PHQ^d^ score, mean (SD; range)	5.4 (4.3; 0-24)	
	PHQ score≥9 (moderate or higher depression), n (%)	30 (15.3)	
	GAD^e^ score, mean (SD; range)	4.6 (3.8; 0-21)	
	GAD score≥9 (moderate or higher anxiety), n (%)	19 (9.7)	
	PAID^f^ score, mean (SD; range)	8.0 (5.5; 0-20)	
	DERS^g^ score, mean (SD; range)	1.9 (0.6; 1-5)	

^a^Possible score ranges for the surveys were listed. Only for the Generalized Anxiety Disorder Scale, the observed score range (0-19) was different from the possible range.

^b^SWLS: Satisfaction with Life Scale.

^c^TIPI: Ten-Item Personality Inventory.

^d^PHQ: Patient Health Questionnaire.

^e^GAD: Generalized Anxiety Disorder Scale.

^f^PAID: Problem Areas in Diabetes Scale assessing diabetes distress.

^g^DERS: Difficulties in Emotion Regulation Scale.

**Table 3:** Summary statistics for ecological momentary assessment (EMA) variables.

EMA variable	All observations, mean (SD; range)	Between-person variance	Average within-person variance	ICC^a^	Between-person variance of log within-person variances
NA^b^ drift rate	–0.05 (0.84; –2.43 to 2.31)	0.19	0.54	0.27	0.27
PA^c^ drift rate	–0.53 (0.83; –1.68 to 2.05)	0.19	0.51	0.28	0.18
NA RT^d^	2.22 (1.70; 0.31 to 24.20)	0.70	1.95	0.26	1.00
PA RT	2.39 (1.83; 0.22 to 28.24)	0.84	2.02	0.29	0.89
Sum of 4 NA items	19.69 (20.26; 0 to 100)	247.10	243.15	0.50	1.60
Sum of 4 PA items	49.78 (23.87; 0 to 100)	338.85	327.01	0.51	1.40
Sum of dichotomized NA items	0.71 (1.21; 0 to 4)	0.75	1.95	0.28	3.27
Sum of dichotomized PA items	2.43 (1.50; 0 to 4)	1.05	1.88	0.36	1.64

^a^ICC: intraclass correlation coefficient.

^b^NA: negative affect.

^c^PA: positive affect.

^d^RT: response time.

### Reliability

Figure S3 in Multimedia Appendix 1 shows the between-person reliabilities as a function of the number of EMA prompts completed for each proposed emotional clarity indicator. For all indicators, reliability increased with more prompts, but each of the proposed emotional clarity indicators demonstrated acceptable reliability (ie, ≥0.70) with a relatively small number of EMA occasions. NA RT required 5 EMA prompts for acceptable reliability, and PA RT required 4 prompts. Both average NA and PA drift rates showed 0.70 reliability when 7 EMA prompts were completed.

### Validity

Relationships between NA RT and other measures were not consistent with our hypotheses. No significant associations were found with subjective well-being or emotion regulation measures (Table 4; *P* values ranging from .08 to .98). PA RT was not significantly associated with any well-being or emotion regulation variable (Table 4; *P* values ranging from 25 to 93), except, unexpectedly, for diabetes distress (*r*=0.17; *P*=.009). After adjustment for processing speed, the associations between both NA RT and PA RT and diabetes distress were unexpectedly significant in a positive direction (*r*=0.14; *P*=.03 and *r*=0.20; *P*=.002, respectively). Adjustment for emotional intensity changed effects very minimally at the between-person level, consistent with the findings of a prior study [17], so the results from that model were not reported here. Both NA RT and PA RT had significant associations with processing speed (*r*=0.25; *P*<.001 and *r*=0.21; *P*=.001, respectively).

The associations between NA drift rate and other measures were consistent with our hypotheses overall. Unexpectedly, NA drift rate was not related to the lack of emotional clarity (*P*=.13) or 3 of the other DERS subscales (Table 4; *P* values from .18 to .76). It was also not associated with life satisfaction (*P*=.17). However, a greater NA drift rate was significantly associated with lower neuroticism (*r*=–0.18; *P*=.01), depression (*r*=–0.27; *P*<.001), anxiety (*r*=–0.27; *P*<.001), and diabetes distress (*r*=–0.17; *P*=.005); lower overall difficulties with emotion regulation (total DERS score; *r*=–0.15; *P*=.03); less limited emotion regulation strategies (*r*=–0.15; *P*=.048); and lower lack of emotion awareness (*r*=–0.18; *P*=.009). Adjustment by processing speed resulted in minimal changes. When NA and PA drift rates were computed from mood items that were dichotomized at each person’s mean NA and PA ratings (rather than at the scale midpoint), the results showed mostly nonsignificant correlations with other measures (refer to Table S3 in Multimedia Appendix 1).

**Table 4:** Between-person correlations between emotional clarity indices and other measures. All correlations were adjusted for the baseline speed of responding and time of day, but only columns with adjusted values additionally had processing speed as a control variable.

						NA^a^ drift rate			NA drift rate, adjusted^b^			PA^c^ drift rate			PA drift rate, adjusted^b^			NA RT^d,e^			NA RT, adjusted^b,e^			PA RT^e^			PA RT, adjusted^b,e^
**Subjective well-being**																											
	**Satisfaction with life**																										
		*r*			0.1			0.1			–0.1			–0.1			–0.03			–0.04			–0.04			–0.05	
		*P* value			.17			.18			.09			.09			.67			.57			.51			.45	
	**Neuroticism**																										
		*r*			–0.18			–0.18			–0.08			–0.08			–0.06			–0.06			–0.01			–0.01	
		*P* value			.01^f^			.01^f^			.29			.29			.51			.44			.91			.85	
	**Depression**																										
		*r*			–0.27			–0.27			0.03			0.03			0			0			0.06			0.06	
		*P* value			<.001^f^			<.001^f^			.73			.73			.98			.99			.47			.45	
	**Anxiety**																										
		*r*			–0.27			–0.27			–0.09			–0.09			–0.09			–0.09			–0.03			–0.03	
		*P* value			<.001^f^			<.001^f^			.21			.22			.34			.28			.77			.74	
	**Diabetes distress**																										
		*r*			–0.17			–0.16			0.17			0.17			0.09			0.14			0.17			0.2	
		*P* value			.005^f^			.008^f^			.009^f^			.008^f^			.14			.03^f^			.009^f^			.002^f^	
**Emotion regulation**																											
	**DERS^g^ (total)**																										
		*r*			–0.15			–0.16			0.03			0.03			0.03			0.01			0.05			0.03	
		*P* value			.03^f^			.01^f^			.78			.78			.64			.92			.39			.58	
		**Limited strategies**																									
			*r*	–0.15			–0.15			0.03			0.03			–0.04			–0.04			–0.03			–0.03		
			*P* value	.048^f^			.04^f^			.76			.76			.59			.59			.66			.67		
		**Nonacceptance**																									
			*r*	–0.1			–0.11			0.04			0.03			–0.01			–0.03			0			–0.01		
			*P* value	.18			.13			.64			.66			.87			.61			.93			.85		
		**Impulse control difficulties**																									
			*r*	0.02			0.03			0.13			0.13			0.02			0.04			0.02			0.05		
			*P* value	.76			.65			.18			.18			.79			.39			.67			.33		
		**Difficulties with goal directedness**																									
			*r*	–0.07			–0.08			–0.11			–0.11			0.11			0.09			0.08			0.06		
			*P* value	.32			.26			.20			.18			.08			.13			.25			.36		
		**Lack of awareness**																									
			*r*	–0.18			–0.18			–0.09			–0.09			–0.07			–0.1			–0.01			–0.03		
			*P* value	.009^f^			.005^f^			.20			.18			.35			.19			.84			.64		
		**Lack of emotional clarity**																									
			*r*	–0.11			–0.12			0.04			0.04			0			–0.03			0.03			0		
			*P* value	.13			.09			.59			.59			.95			.64			.73			.95		
	**Processing speed (control variable)**																										
		*r*			0.09			—^h^			0.02			—			0.25			—			0.21			—	
		*P* value			.18			—			.78			—			<.001^f^			—			<.001^f^			—	

^a^NA: negative affect.

^b^Adjusted for processing speed.

^c^PA: positive affect.

^d^RT: response time.

^e^Multiplied by –1 so that higher values indicate greater emotional clarity.

^f^*P*<.05.

^g^DERS: Difficulties in Emotion Regulation Scale.

^h^Not applicable.

Consistent with expectations, PA drift rate was not significantly associated with most subjective well-being or NA-specific emotion regulation measures (Table 4; *P* values from .09 to .76). Unexpectedly, PA drift rate was not significantly associated with emotional clarity (*P*=.59) or emotional awareness (*P*=.20) before or after adjustment for processing speed. Furthermore, contrary to expectations, a greater PA drift rate was associated with greater diabetes distress (*r*=0.17; *P*=.009). Neither NA drift rate nor PA drift rate had significant associations with processing speed (*P*=.18 and *P*=.78, respectively). Without adjustment for baseline speed, a few of the associations differed, such as the relationship between NA drift rate and life satisfaction (*r*=0.16; *P*=.02; Table 5).

**Table 5:** Between-person correlations between emotional clarity indices and other measures. Correlations were adjusted for the time of day but not for the baseline speed of responding, and only columns with adjusted values had processing speed as a control variable.

						NA^a^ drift rate			NA drift rate, adjusted^b^			PA^c^ drift rate			PA drift rate, adjusted^b^			NA RT^d,e^			NA RT, adjusted^b,e^			PA RT^e^			PA RT, adjusted^b,e^
**Subjective well-being**																											
	**Satisfaction with life**																										
		*r*			0.16			0.14			0			–0.03			0.1			0.06			0.09			0.05	
		*P* value			.02^f^			.05			.96			.67			.15			.40			.15			.42	
	**Neuroticism**																										
		*r*			–0.14			–0.16			–0.05			–0.06			–0.01			–0.04			0.02			0	
		*P* value			.04^f^			.02^f^			.48			.38			.87			.63			.74			.94	
	**Depression**																										
		*r*			–0.27			–0.28			–0.01			0			–0.05			–0.04			–0.01			0	
		*P* value			<.001^f^			<.001^f^			.92			1.00			.43			.57			.85			.95	
	**Anxiety**																										
		*r*			–0.25			–0.26			–0.08			–0.09			–0.07			–0.08			–0.03			–0.03	
		*P* value			<.001^f^			<.001^f^			.24			.21			.34			.24			.69			.64	
	**Diabetes distress**																										
		*r*			–0.25			–0.2			0.04			0.1			–0.08			0.03			–0.04			0.07	
		*P* value			<.001^f^			.003^f^			.53			.17			.23			.67			.59			.33	
**Emotion regulation**																											
	**DERS^g^ (total)**																										
		*r*			–0.15			–0.17			0			–0.01			–0.01			–0.04			0			–0.02	
		*P* value			.045^f^			.009^f^			.97			.92			.91			.51			.95			.70	
		**Limited strategies**																									
			*r*	–0.12			–0.13			0.03			0.04			–0.02			–0.02			–0.01			–0.01		
			*P* value	.12			.07			.70			.67			.81			.78			.90			.90		
		**Nonacceptance**																									
			*r*	–0.06			–0.09			0.06			0.04			0.04			–0.01			0.05			0		
			*P* value	.41			.18			.42			.61			.58			.83			.44			.95		
		**Impulse control difficulties**																									
			*r*	0			0.03			0.09			0.12			–0.02			0.04			–0.02			0.04		
			*P* value	.99			.67			.36			.21			.79			.48			.82			.47		
		**Difficulties with goal directedness**																									
			*r*	–0.07			–0.09			–0.1			–0.12			0.07			0.04			0.04			0.01		
			*P* value	.35			.21			.23			.14			.27			.46			.47			.79		
		**Lack of awareness**																									
			*r*	–0.19			–0.21			–0.12			–0.13			–0.1			–0.14			–0.06			–0.09		
			*P* value	.01^f^			.002^f^			.13			.07			.13			.02^f^			.32			.12		
		**Lack of emotional clarity**																									
			*r*	–0.14			–0.16			–0.01			–0.02			–0.07			–0.1			–0.05			–0.07		
			*P* value	.07			.04^f^			.92			.81			.38			.17			.54			.32		
	**Processing speed (control variable)**																										
		*r*			0.28			—^h^			0.25			—			0.5			—			0.48			—	
		*P* value			<.001^f^			—			.005^f^			—			<.001^f^			—			<.001^f^			—	

^a^NA: negative affect.

^b^Adjusted for processing speed.

^c^PA: positive affect.

^d^RT: response time.

^e^Multiplied by –1 so that higher values indicate greater emotional clarity.

^f^*P*<.05.

^g^DERS: Difficulties in Emotion Regulation Scale.

^h^Not applicable.

## Discussion

### Principal Findings

The most notable finding from this study was that the average NA drift rate, a proposed indicator of typical emotional clarity, had expected associations overall with validated measures of subjective well-being and emotion regulation, both before and after adjustment for processing speed and emotional intensity. By contrast, NA RT, another proposed indicator of typical emotional clarity, did not have the anticipated associations with the validated measures. Relative to NA drift rate, NA RT may be confounded by a greater number of factors aside from emotional clarity.

Nevertheless, there is no sufficient evidence to conclude that the average NA drift rate is a valid indicator of NA clarity, while the average NA RT is not a valid indicator of NA clarity due to sample size constraints. In post hoc power analyses, with our sample size of 196 participants, there was 80% power to detect a between-person correlation of 0.20. Therefore, the study may have been underpowered to detect small correlations that might still be meaningful. Furthermore, the sample size was not chosen a priori to be sufficiently powered to detect between-person relationships after adjustment for multiplicity of testing [59] because the sample size was conditioned on research questions that were more primary for the diabetes EMA study.

While we cannot conclude that NA drift rate is a valid indicator of NA clarity, our results suggest that researchers in future studies should continue to investigate NA drift rate as an implicit measure of emotional clarity. We applied stringent validity tests by adjusting for both individual differences in the baseline speed of responding and processing speed and found significant associations between NA drift rate and 4 (80%) of the 5 subjective well-being ratings. Correlations with 4 (67%) of the 6 emotion regulation subscales were in the expected directions. Finally, it is notable that NA drift rate had a correlation of 0.63 (*P*<.001) with NA RT. People with a higher NA drift rate responded faster to the NA emotion items, had greater subjective well-being (eg, fewer depression symptoms), and had fewer difficulties with overall emotion regulation, which are all aligned with the expectation for an emotional clarity measure. Collectively, study results suggest that NA drift rate deserves further attention in future research.

Some of the magnitudes of correlations between NA drift rate and other study measures were comparable to sizes of correlations between formal (self-report) assessments of emotional clarity and other measures found in prior studies. One study found that in a group with generalized anxiety disorder, a self-report assessment of emotional clarity had correlations of –0.29 and –0.33 with depression and anxiety, respectively [60], which are similar to the correlations found in this study. In the same study, the association between emotional clarity and depression or anxiety was not significant in the healthy control group [60], suggesting that the mental health status of the sample may affect the magnitude of the observed relationships. Other correlations found between emotional clarity and depression were –0.24 in a clinical sample [61] and –0.29 in elementary school–aged children [62]. Correlations between self-reported emotional clarity and neuroticism ranged between –0.31 in a sample of adolescents [63] and –0.37 in a sample of college students [64], and correlations between emotional clarity and life satisfaction ranged between 0.31 in adolescents [65] and 0.35 in undergraduate students [36], which are larger than the correlations observed in this study.

When NA drift rate was computed from mood items that were dichotomized at each person’s mean response rather than the scale midpoint, no significant associations were observed with other study measures. The distance-difficulty hypothesis underlying the D-diffusion model that was used to generate the NA drift parameter assumes that people respond faster to items that contrast more with their current state [23]. According to this hypothesis, an individual with very low NA (regardless of their average level of NA) was expected to quickly report not being scared (an item often associated with high NA). Dichotomizing mood at each person’s own midpoint created a variable representing whether their mood was higher or lower relative to their personal average and not higher or lower in absolute terms (which could be approximated by dichotomizing at the scale midpoint). Perhaps, the former was less relevant to the distance-difficulty hypothesis compared to the latter because it captured relative mood and not actual mood, leading to the creation of NA drift parameters with no associations with other study measures.

The reliabilities for the average NA drift rate, PA drift rate, NA RT, and PA RT were all acceptable with a small number of EMA prompts (ie, 4 to 7 EMA prompts or 1 to 2 days of EMA surveys). Therefore, reliable measurement of these proposed emotional clarity indicators would likely be feasible in most EMA studies where affect items are administered and RTs are recorded.

### Secondary Findings

We found preliminary evidence supporting the argument that emotional clarity deficits are valence specific [9]. NA drift rate and PA drift rate had differential associations with self-report measures. Furthermore, they were moderately correlated with each other (*r*=0.38; refer to Table S1 in Multimedia Appendix 1). Had NA and PA drift rates been redundant with one another, a high correlation would have been expected.

Greater PA drift rate and PA RT were unexpectedly found to be associated with higher diabetes distress and not associated with the awareness or clarity subscales of the DERS. It is unclear why people with greater diabetes distress would have greater clarity of positive emotions. Perhaps, when overwhelmed with burden from diabetes, people had a greater appreciation of positive emotional states and hence greater clarity of PA. One possible reason why PA drift rate and PA RT had far from significant associations with self-reported emotional clarity may have been because, given that other items in the DERS-SF asked questions relevant to the NA context, participants were primed to answer the emotional clarity questions with reference to feeling NA. More assessments of the validity of PA drift rate and PA RT are needed.

### Future Directions

More conclusive evidence of the validity of RT-based measures of emotional clarity may come from studies where NA clarity can be manipulated, and the proposed emotional clarity indicators can be compared for sensitivity to these changes. For instance, people who undergo a mindfulness intervention may be expected to have higher NA clarity in the period following the intervention, and this effect should be reflected in changes in drift rates or RTs to NA EMA items.

EMA mood item RT–based measures of emotional clarity have a great potential utility. They can serve as indices of emotional clarity that do not require burdening participants with additional emotional clarity items. Furthermore, they can help avoid possible issues with subjective reports, including poor insight into emotional clarity and the possibility of social desirability bias [12]. Although the validity of the RT-based emotional clarity measures at the within-person level was not investigated in this study, such validity would allow for the investigation of changes in emotional clarity within and across days and the situational factors that contribute to them. For the potential utility of EMA mood item RT–based measures of emotional clarity to be realized, further investigations of the validity of EMA RT–based measures of emotional clarity (ie, at both the between-person and within-person levels) are needed.

### Limitations

We had decided not to adjust for multiple comparisons, but to also acknowledge that any results would require replication by future studies. It has been argued that the need for adjustment for multiple comparisons should be evaluated on a case-by-case basis and *P* value adjustment should not be used for all analyses [66]. For instance, adjustment for multiple comparisons comes with not only the benefit of lowering type 1 error but also the disadvantage of increasing the chance of type 2 error. Therefore, one factor to consider when deciding whether to adjust for multiple comparisons is the relative cost of type 1 and type 2 errors for a particular research question [67]. In confirmatory studies with results that have implications for changes in clinical practice or the use of a treatment, the cost of a type 1 error may be higher than that of a type 2 error; hence, *P* value adjustment for multiple comparisons would be sensible [66]. When performing post hoc analyses on existing data as part of theory building and testing (and without direct treatment implications), the relative cost of a type 2 error may be higher; hence, there may be a stronger argument for not using multiple comparisons adjustment [66]. That is, a type 2 error could cause researchers to not detect potentially important findings [68]. If adjustment is not used, there would need to be an acknowledgment that, to account for the possibility of a type 1 error, further research is needed to examine whether results can be replicated [66].

Nevertheless, we still tested the effect of false discovery rate adjustment on the *P* values of correlations for the different groups of hypotheses that were tested (eg, association between NA drift rate and subjective well-being measures). Adjustments for multiple comparisons are often applied separately for distinct families of hypotheses [69]. Tables S4 and S5 in Multimedia Appendix 1 show the false discovery rate–adjusted *P* values associated with Tables 4 and 5, respectively. The biggest differences to note were that several of the associations between NA drift rate and emotional regulation measures were no longer significant.

Using the drift-diffusion model had the advantage of reducing the impact of individual differences in response caution (and potential careless responding) from emotion item RTs but had the disadvantage of assuming a 2-choice task (eg, a high vs low NA) underlying people’s emotion ratings. Because the drift-diffusion model required making the continuous PA and NA items dichotomous for data analysis, granular differences in emotional clarity may have been missed with the drift rate parameter.

We were unable to examine the within-person validity of RT-based clarity measures because EMA measures key to such testing (eg, self-reported emotional clarity and mood regulation success) [12] were not administered in this study. Future studies are needed to examine the within-person validity of the drift rate parameter as a within-person indicator of emotional clarity.

The context in which RTs for emotion items were calculated was similar but not identical to that in prior work. For instance, in this study, median RTs for EMA were calculated based on RTs to 4 items. In prior studies, the median RTs for 5 to 8 items were computed [12,17]. In the original paper examining the validity of RTs to emotion items as indicators of emotional clarity, bipolar mood items were used (eg, items with options from “very unhappy” to “very happy”) [12], whereas our study analyzed unipolar mood items. The results of this study may have been impacted to an extent by differences in EMA administration, such as variations in the type of emotion items used.

Additional evidence is needed that study results generalize beyond adults with T1D. Other populations with an increased likelihood of experiencing lower subjective well-being (eg, individuals with various chronic conditions) [70] may be appropriate targets for future emotional clarity studies.

### Conclusions

A measure of NA drift rate derived from RTs to momentary NA items had expected associations with validated measures of relevance to emotional clarity, providing initial evidence supporting its validity as an indicator of individual differences in the clarity of negative emotions. The validities of NA RT, PA RT, and PA drift rate were not strongly supported by our results. More studies are needed to investigate the validities of NA and PA drift rate and NA and PA RT with larger sample sizes. The development of passive measures of emotional clarity would help create minimally burdensome measures of emotional clarity that are less vulnerable to possible issues from subjective self-reports, such as poor clarity insight and social desirability bias. Such measures may be useful in investigations relevant to the role of emotional clarity in people’s experience of well-being.

## Appendix

Multimedia Appendix 1 Plots of between person reliability as a function of number of ecological momentary assessments and unadjusted between person correlations between study variables.

## Abbreviations

CFI: comparative fit index
DERS: Difficulties in Emotion Regulation Scale
DERS-SF: Difficulties in Emotion Regulation Scale short form
EMA: ecological momentary assessment
IRT: item response theory
MSEM: multilevel structural equation model
NA: negative affect
PA: positive affect
RMSEA: root mean square error of approximation
RT: response time
SRMR: standardized root mean square residual
T1D: type 1 diabetes
TLI: Tucker-Lewis Index

## Data Availability

The data set analyzed for this study is available from the corresponding author, RH, on reasonable request.
