# Supplementary material for: Reliability and Validity of Ecological Momentary Assessment Response Time–Based Measures of Emotional Clarity: Secondary Data Analysis
Source: JMIR Ment Health. 2024 Jul 18;11:e58352. doi: 10.2196/58352 (PMC11294766; doi:10.2196/58352)
Supplement: Multimedia Appendix 1 [file mental_v11i1e58352_app1.docx]

**Appendix 1**

**Figure S1.** Histograms of the predicted and observed response time (RT) distributions for the D-diffusion model applied to the negative affect items: Item 1 = tense, item 2 = upset, item 3 = sad, item 4 = disappointed. Bars on the histograms are the observed RTs while dotted lines are predicted RTs.


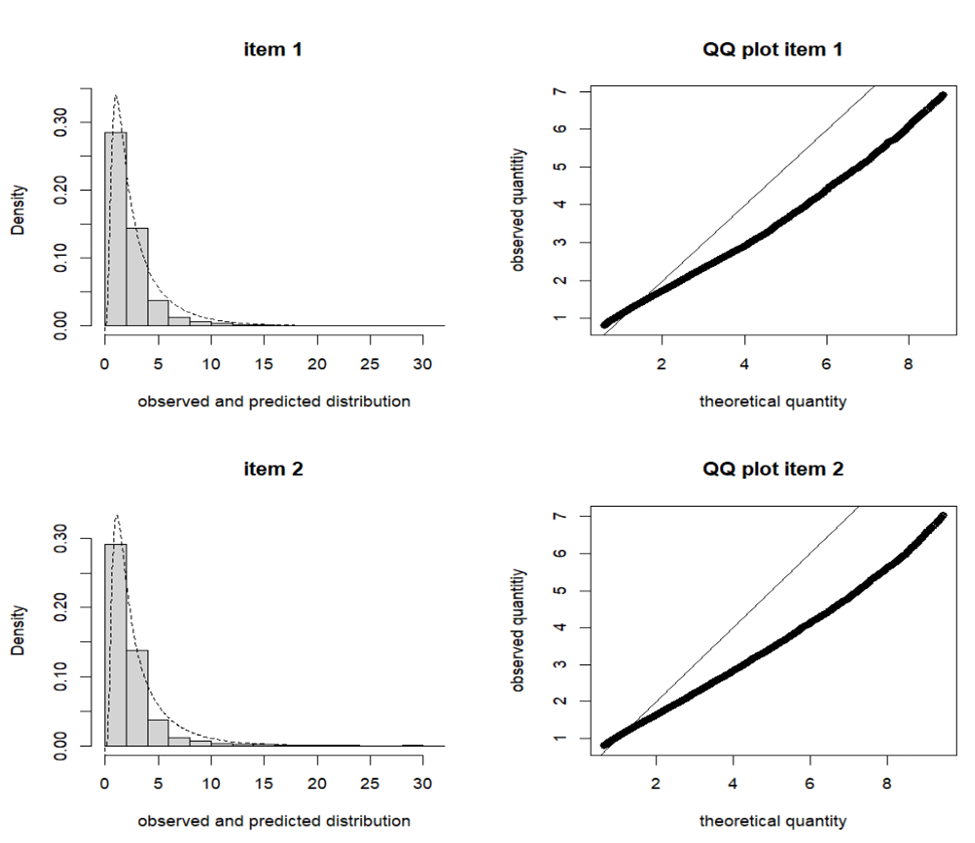

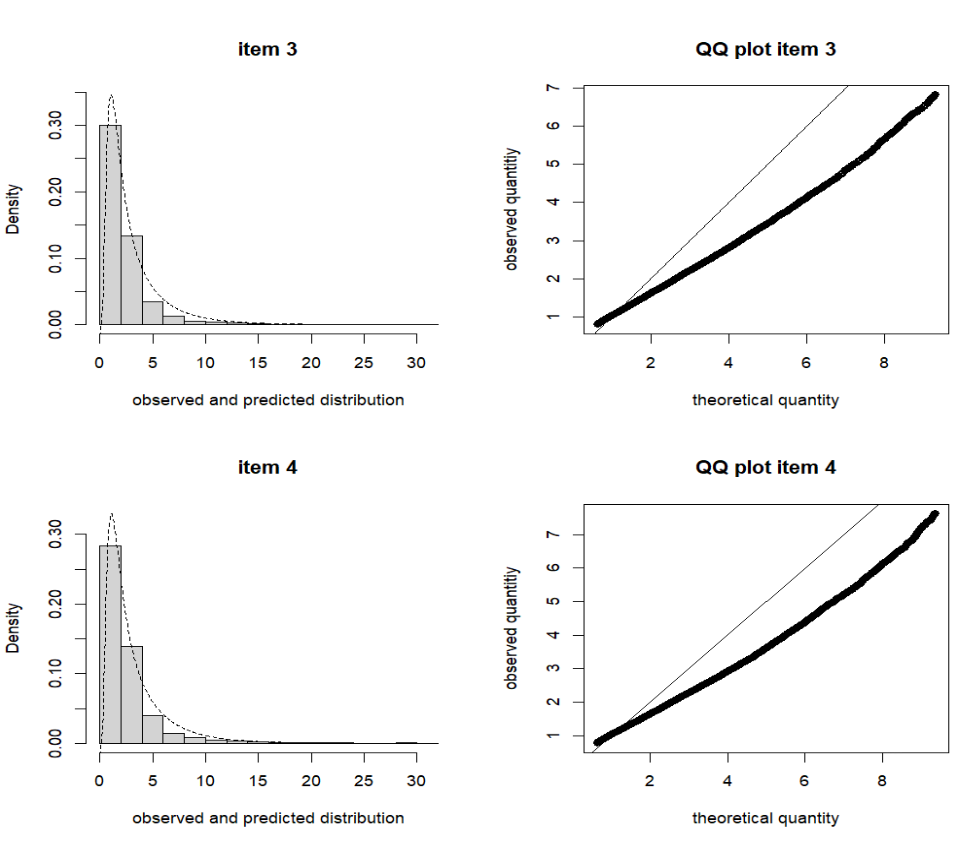


**Figure S2.** Histograms of the predicted and observed response time distributions for the D-diffusion model applied to the positive affect items: Item 1 = happy, item 2 = content, item 3 = enthusiastic, item 4 = excited. Bars on the histograms are the observed RTs while dotted lines are predicted RTs.


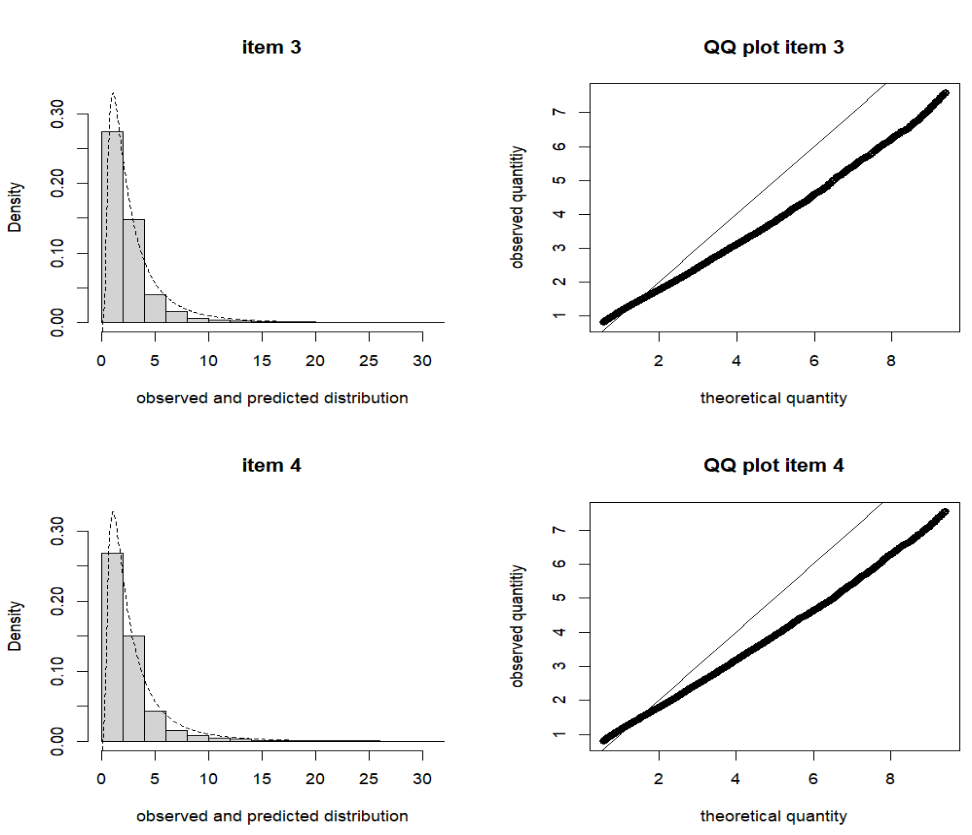

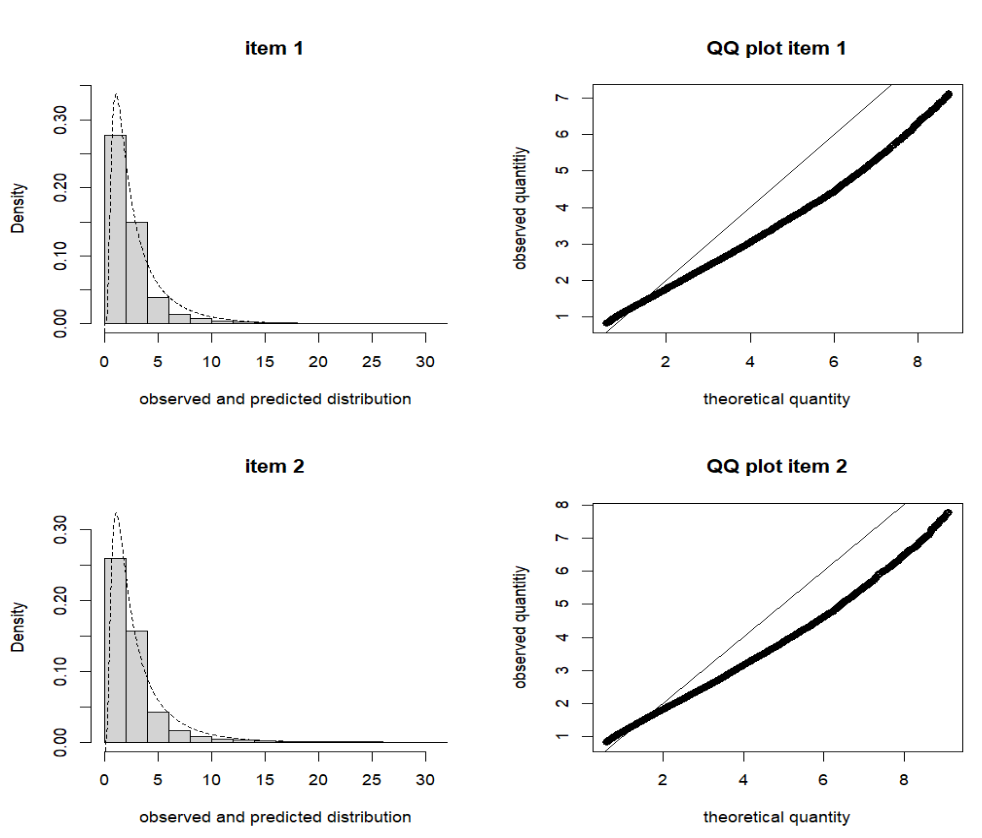


**Figure S3.** The plots depict: a) Between-person reliability of negative affect drift rate based on number of ecological momentary assessments; b) Between-person reliability of positive affect drift rate based on number of ecological momentary assessments; c) Between-person reliability of (median) negative affect response times based on number of ecological momentary assessments; d) Between-person reliability of (median) positive affect response times based on number of ecological momentary assessments.

a) Negative affect drift rate reliability


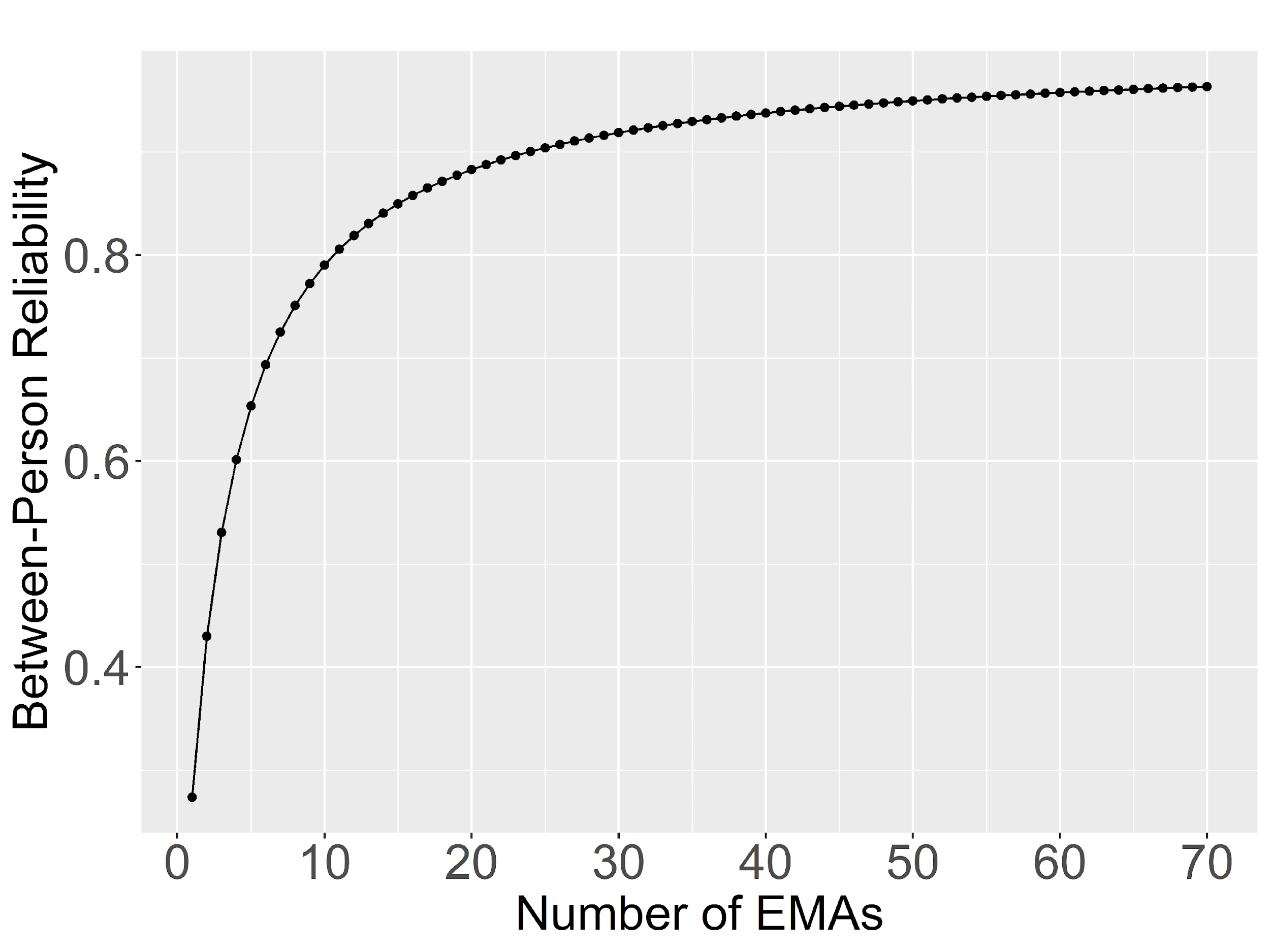

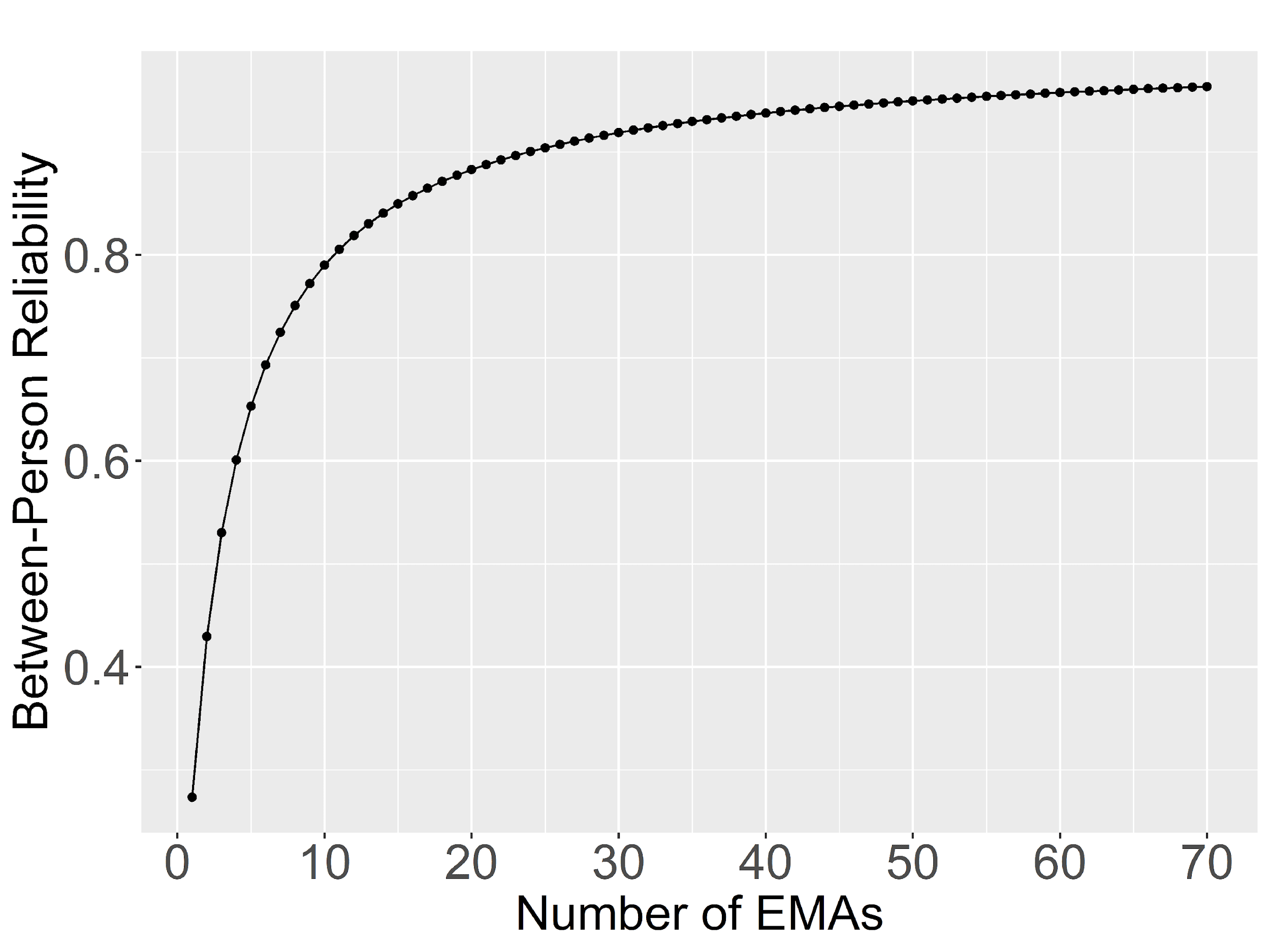


b) Positive affect drift rate reliability

c) Negative affect response time reliability

d) Positive affect response time reliability


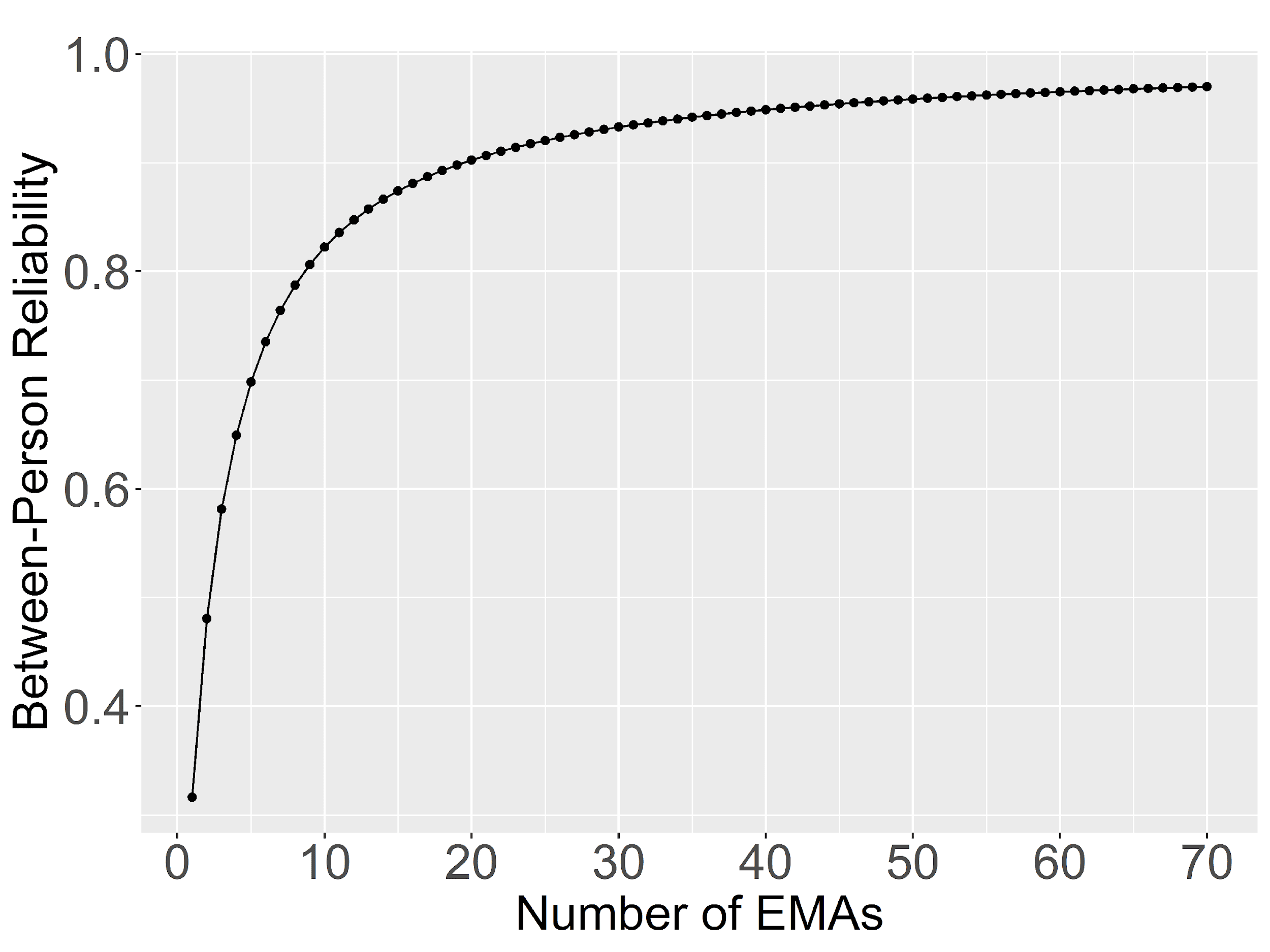


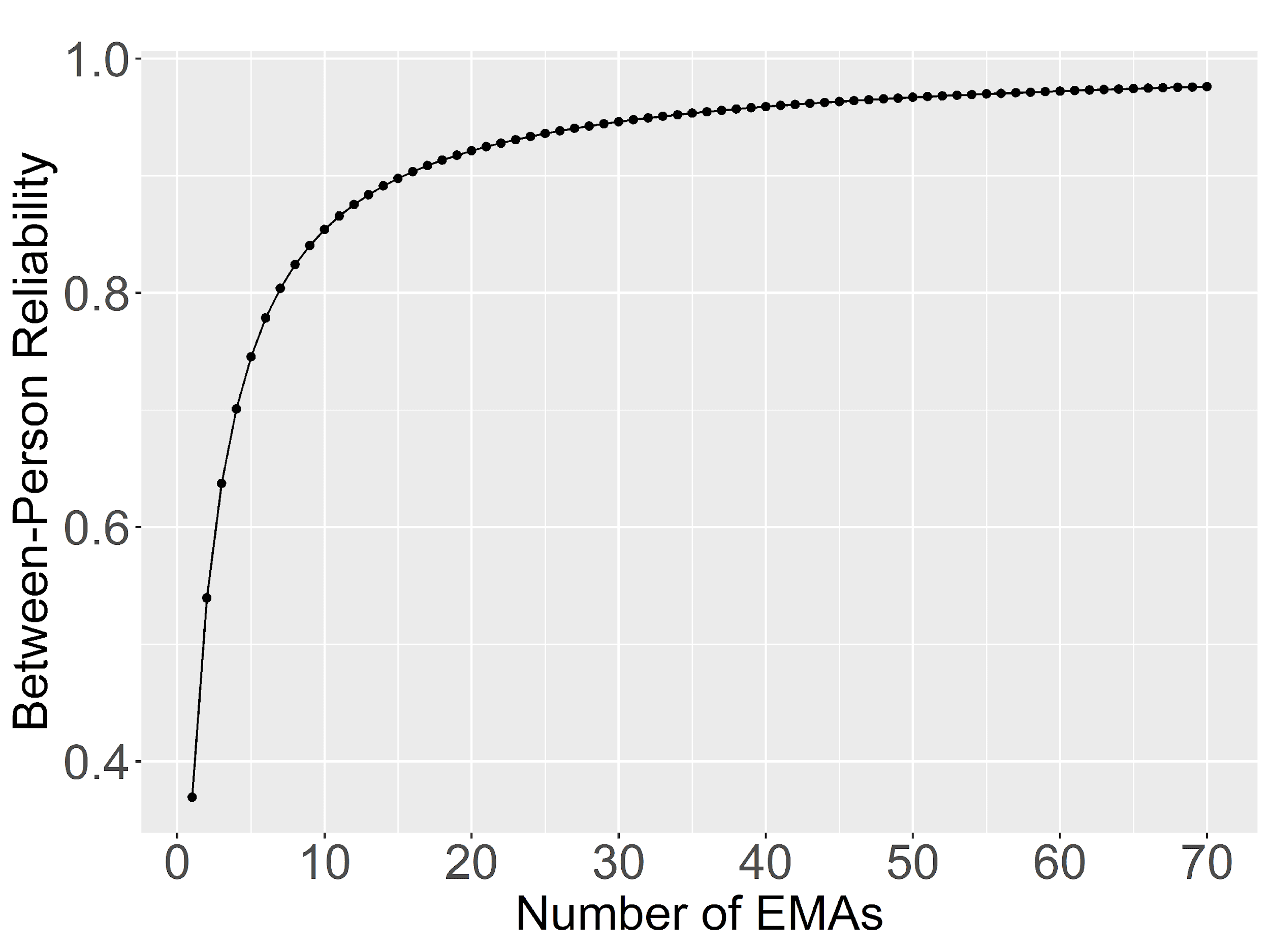


**Table S1.** Unadjusted between-person correlations between RT based emotional clarity indicators and the Difficulties in Emotion regulation Scale, and its subscales.

|  | NA Drift | PA Drift | NA RT^b^ | PA RT^b^ | DERS (total) | Strategies | Non-accept. | Impulse | Goals | Aware-ness | Clarity |
| --- | --- | --- | --- | --- | --- | --- | --- | --- | --- | --- | --- |
| NA Drift | 1 |  |  |  |  |  |  |  |  |  |  |
| PA Drift | 0.38 (*P*<.001)^a^ | 1 |  |  |  |  |  |  |  |  |  |
| NA RT^b^ | 0.63 (*P*<.001)^a^ | 0.51 (*P*<.001)^a^ | 1 |  |  |  |  |  |  |  |  |
| PA RT^b^ | 0.5 (*P*<.001)^a^ | 0.56 (*P*<.001)^a^ | 0.96 (*P*<.001)^a^ | 1 |  |  |  |  |  |  |  |
| DERS (total) | -0.15 (*P*=.043)^a^ | -0.01 (*P*=.935) | -0.01 (*P*=.883) | 0 (*P*=.98) | 1 |  |  |  |  |  |  |
| Limited Strategies | -0.12 (*P*=.113) | 0.02 (*P*=.778) | -0.02 (*P*=.785) | -0.02 (*P*=.813) | 0.79 (*P*<.001)^a^ | 1 |  |  |  |  |  |
| Non-acceptance | -0.06 (*P*=.429) | 0.06 (*P*=.412) | 0.04 (*P*=.604) | 0.05 (*P*=.471) | 0.59 (*P*<.001)^a^ | 0.37 (*P*<.001)^a^ | 1 |  |  |  |  |
| Impulse control difficulties | 0 (*P*=.949) | 0.08 (*P*=.442) | -0.02 (*P*=.747) | -0.03 (*P*=.714) | 0.67 (*P*<.001)^a^ | 0.53 (*P*<.001)^a^ | 0.2 (*P*=.001)^a^ | 1 |  |  |  |
| Difficulties with goal directedness | -0.07 (*P*=.298) | -0.12 (*P*=.151) | 0.07 (*P*=.31) | 0.04 (*P*=.544) | 0.74 (*P*<.001)^a^ | 0.64 (*P*<.001)^a^ | 0.36 (*P*<.001)^a^ | 0.44 (*P*<.001)^a^ | 1 |  |  |
| Lack of Awareness | -0.19 (*P*=.009)^a^ | -0.13 (*P*=.08) | -0.11 (*P*=.119) | -0.07 (*P*=.293) | 0.41 (*P*<.001)^a^ | 0.18 (*P*=.023)^a^ | 0.06 (*P*=.422) | 0.18 (*P*=.022)^a^ | 0.06 (*P*=.435) | 1 |  |
| Lack of Clarity | -0.14 (*P*=.073) | -0.01 (*P*=.922) | -0.07 (*P*=.387) | -0.04 (*P*=.569) | 0.64 (*P*<.001)^a^ | 0.34 (*P*<.001)^a^ | 0.3 (*P*<.001)^a^ | 0.31 (*P*<.001)^a^ | 0.26 (*P*<.001)^a^ | 0.47 (*P*<.001)^a^ | 1 |

DERS-Difficulties in Emotion regulation Scale; NA-negative affect; PA-positive affect; RT-response time

^a^<.05

^b^Multiplied by -1 so that higher values indicate greater emotional clarity

**Table S2.** Unadjusted between-person correlations between RT based emotional clarity indicators and other study measures.

|  | NA Drift | PA Drift | NA RT^b^ | PA RT^b^ | SWLS | Neuro | PHQ | GAD | PAID | SS | NA | PA | BSL RT |
| --- | --- | --- | --- | --- | --- | --- | --- | --- | --- | --- | --- | --- | --- |
| NA Drift | 1 |  |  |  |  |  |  |  |  |  |  |  |  |
| PA Drift | 0.37 (*P*<.001)^a^ | 1 |  |  |  |  |  |  |  |  |  |  |  |
| NA RT^b^ | 0.63 (*P*<.001)^a^ | 0.51 (*P*<.001)^a^ | 1 |  |  |  |  |  |  |  |  |  |  |
| PA RT^b^ | 0.49 (*P*<.001)^a^ | 0.56 (*P*<.001)^a^ | 0.96 (*P*<.001)^a^ | 1 |  |  |  |  |  |  |  |  |  |
| SWLS | 0.16 (*P*=.028)^a^ | -0.01 (*P*=.859) | 0.1 (*P*=.168) | 0.09 (*P*=.16) | 1 |  |  |  |  |  |  |  |  |
| Neuro | -0.13 (*P*=.068) | -0.05 (*P*=.454) | -0.01 (*P*=.845) | 0.02 (*P*=.752) | -0.18 (*P*=.009)^a^ | 1 |  |  |  |  |  |  |  |
| PHQ | -0.27 (*P*<.001)^a^ | 0 (*P*=.961) | -0.05 (*P*=.442) | -0.01 (*P*=.836) | -0.36 (*P*<.001)^a^ | 0.44 (*P*<.001)^a^ | 1 |  |  |  |  |  |  |
| GAD | -0.24 (*P*=.001)^a^ | -0.09 (*P*=.234) | -0.07 (*P*=.348) | -0.03 (*P*=.672) | -0.3 (*P*<.001)^a^ | 0.5 (*P*<.001)^a^ | 0.74 (*P*<.001)^a^ | 1 |  |  |  |  |  |
| PAID | -0.24 (*P*<.001)^a^ | 0.04 (*P*=.529) | -0.08 (*P*=.219) | -0.04 (*P*=.585) | -0.38 (*P*<.001)^a^ | 0.24 (*P*=.001)^a^ | 0.5 (*P*<.001)^a^ | 0.45 (*P*<.001)^a^ | 1 |  |  |  |  |
| SS | 0.27 (*P*<.001)^a^ | 0.24 (*P*=.006)^a^ | 0.5 (*P*<.001)^a^ | 0.47 (*P*<.001)^a^ | 0.1 (*P*=.179) | 0.04 (*P*=.552) | -0.03 (*P*=.734) | 0 (*P*=.976) | -0.21 (*P*=.003)^a^ | 1 |  |  |  |
| NA | -0.53 (*P*<.001)^a^ | 0.19 (*P*=.018)^a^ | -0.04 (*P*=.622) | 0.1 (*P*=.112) | -0.2 (*P*=.008)^a^ | 0.25 (*P*<.001)^a^ | 0.38 (*P*<.001)^a^ | 0.35 (*P*<.001)^a^ | 0.39 (*P*<.001)^a^ | -0.01 (*P*=.881) | 1 |  |  |
| PA | 0.08 (*P*=.254) | 0.21 (*P*=.014)^a^ | -0.11 (*P*=.056) | -0.09 (*P*=.127) | 0.3 (*P*<.001)^a^ | -0.28 (*P*<.001)^a^ | -0.23 (*P*=.002)^a^ | -0.17 (*P*=.005)^a^ | -0.1 (*P*=.197) | -0.26 (*P*<.001)^a^ | -0.07 (*P*=.415) | 1 |  |
| BSL RT | -0.41 (*P*<.001)^a^ | -0.5 (*P*<.001)^a^ | -0.71 (*P*<.001)^a^ | -0.73 (*P*<.001)^a^ | -0.18 (*P*=.005)^a^ | -0.04 (*P*=.535) | 0.06 (*P*=.279) | 0.02 (*P*=.788) | 0.21 (*P*=.001)^a^ | -0.46 (*P*<.001)^a^ | -0.02 (*P*=.796) | 0.09 (*P*=.137) | 1 |

BSL RT- Baseline response time; GAD- Generalized Anxiety Disorder; NA-negative affect; Neuro-neuroticism; PA-positive affect; PAID- Problem Areas in Diabetes Scale, diabetes distress; PHQ-Patient Health Questionnaire; RT-response time; SS-Symbol Search (processing speed); SWLS-Satisfaction with Life Scale

^a^p<.05

^b^Multiplied by -1 so that higher values indicate greater emotional clarity

**Table S3.** Between-person correlations between emotional clarity indices and other measures, with the drift rate parameters computed from emotion items dichotomized at each person’s midpoint (i.e., an item was coded as 1 if greater than or equal to a person’s average, and 0 otherwise). All correlations were adjusted for baseline speed of responding, but only columns with “adjusted” additionally had processing speed as a control variable.

|  | NA Drift Rate | NA Drift Rate, adjusted^b^ | PA Drift Rate | PA Drift Rate, adjusted^b^ |
| --- | --- | --- | --- | --- |
| **Subjective well-being** | | | | |
| Satisfaction with Life | -.1 (*P*=.147) | -.1 (*P*=.131) | -.05 (*P*=.465) | -.06 (*P*=.436) |
| Neuroticism | -.1 (*P*=.13) | -.11 (*P*=.118) | -.06 (*P*=.43) | -.07 (*P*=.41) |
| Depression | -.08 (*P*=.259) | -.08 (*P*=.262) | .02 (*P*=.815) | .02 (*P*=.81) |
| Anxiety | -.07 (*P*=.311) | -.07 (*P*=.297) | .01 (*P*=.849) | .01 (*P*=.844) |
| Diabetes distress | .05 (*P*=.468) | .06 (*P*=.363) | .17 (*P*=.007)^a^ | .19 (*P*=.005)^a^ |
|  |  |  |  |  |
| DERS (total) | .06 (*P*=.384) | .06 (*P*=.432) | .12 (*P*=.142) | .11 (*P*=.169) |
| 1.Limited Strategies | .04 (*P*=.537) | .05 (*P*=.515) | .05 (*P*=.521) | .06 (*P*=.505) |
| 2.Non-acceptance | .07 (*P*=.348) | .06 (*P*=.396) | .14 (*P*=.087) | .13 (*P*=.108) |
| 3.Impulse control difficulties | .1 (*P*=.157) | .1 (*P*=.12) | .1 (*P*=.117) | .11 (*P*=.096) |
| 4.Difficulties with goal directedness | .04 (*P*=.583) | .03 (*P*=.654) | .04 (*P*=.568) | .04 (*P*=.631) |
| 5.Lack of  Awareness | -.08 (*P*=.323) | -.08 (*P*=.264) | 0 (*P*=.945) | 0 (*P*=.979) |
| 6.Lack of emotional  clarity | .06 (*P*=.404) | .06 (*P*=.464) | .09 (*P*=.203) | .08 (*P*=.246) |
| Processing Speed (control variable) | .08 (*P*=.266) | - | .09 (*P*=.264) | - |

DERS- Difficulties in Emotion Regulation Scale; NA-negative affect; PA-positive affect; RT-response time;

^a^p<.05

^b^Adjusted for processing speed

**Table S4.** Between-person correlations between emotional clarity indices and other measures, with p-values adjusted for false discovery rate (counterpart to Table 4). All correlations were adjusted for baseline speed of responding, but only columns with “adjusted” additionally had processing speed as a control variable.

|  | NA Drift Rate | NA Drift Rate, adjusted^b^ | PA Drift Rate | PA Drift Rate, adjusted^b^ | NA RT^c^ | NA RT, adjusted^b,c^ | PA RT^c^ | PA RT, adjusted^b,c^ |
| --- | --- | --- | --- | --- | --- | --- | --- | --- |
| **Subjective well-being** | | | | | | | | |
| Satisfaction with Life | .1 (*P*=.174) | .1 (*P*=.18) | -.1 (*P*=.23) | -.1 (*P*=.232) | -.03 (*P*=.841) | -.04 (*P*=.712) | -.04 (*P*=.855) | -.05 (*P*=.757) |
| Neuroticism | -.18 (*P*=.014)^a^ | -.18 (*P*=.013)^a^ | -.08 (*P*=.368) | -.08 (*P*=.36) | -.06 (*P*=.841) | -.06 (*P*=.712) | -.01 (*P*=.906) | -.01 (*P*=.854) |
| Depression | -.27 (*P*<.001)^a^ | -.27 (*P*<.001)^a^ | .03 (*P*=.725) | .03 (*P*=.725) | 0 (*P*=.982) | 0 (*P*=.985) | .06 (*P*=.855) | .06 (*P*=.757) |
| Anxiety | -.27 (*P*<.001)^a^ | -.27 (*P*<.001)^a^ | -.09 (*P*=.353) | -.09 (*P*=.348) | -.09 (*P*=.841) | -.09 (*P*=.71) | -.03 (*P*=.906) | -.03 (*P*=.854) |
| Diabetes distress | -.17 (*P*=.008)^a^ | -.16 (*P*=.013)^a^ | .17 (*P*=.045)^a^ | .17 (*P*=.04)^a^ | .09 (*P*=.7) | .14 (*P*=.15) | .17 (*P*=.045)^a^ | .2 (*P*=.01)^a^ |
|  | | | | | | | | |
| DERS (total) | -.15 (*P*=.104) | -.16 (*P*=.049)^a^ | .03 (*P*=.777) | .03 (*P*=.779) | .03 (*P*=.945) | .01 (*P*=.917) | .05 (*P*=.934) | .03 (*P*=.942) |
| 1.Limited Strategies | -.15 (*P*=.128) | -.15 (*P*=.098) | .03 (*P*=.777) | .03 (*P*=.779) | -.04 (*P*=.945) | -.04 (*P*=.747) | -.03 (*P*=.934) | -.03 (*P*=.942) |
| 2.Non-acceptance | -.1 (*P*=.235) | -.11 (*P*=.188) | .04 (*P*=.777) | .03 (*P*=.779) | -.01 (*P*=.945) | -.03 (*P*=.747) | 0 (*P*=.934) | -.01 (*P*=.949) |
| 3.Impulse control difficulties | .02 (*P*=.764) | .03 (*P*=.65) | .13 (*P*=.536) | .13 (*P*=.429) | .02 (*P*=.945) | .04 (*P*=.747) | .02 (*P*=.934) | .05 (*P*=.942) |
| 4.Difficulties with goal directedness | -.07 (*P*=.369) | -.08 (*P*=.305) | -.11 (*P*=.536) | -.11 (*P*=.429) | .11 (*P*=.328) | .09 (*P*=.679) | .08 (*P*=.934) | .06 (*P*=.942) |
| 5.Lack of  Awareness | -.18 (*P*=.072) | -.18 (*P*=.035)^a^ | -.09 (*P*=.536) | -.09 (*P*=.429) | -.07 (*P*=.923) | -.1 (*P*=.679) | -.01 (*P*=.934) | -.03 (*P*=.942) |
| 6.Lack of emotional  clarity | -.11 (*P*=.235) | -.12 (*P*=.164) | .04 (*P*=.777) | .04 (*P*=.779) | 0 (*P*=.945) | -.03 (*P*=.747) | .03 (*P*=.934) | 0 (*P*=.949) |
| Processing Speed (control variable) | .09 (*P*=.235) | - | .02 (*P*=.777) | - | .25 (*P*<.001)^a^ | - | .21 (*P*=.008)^a^ | - |

DERS- Difficulties in Emotion Regulation Scale; NA-negative affect; PA-positive affect; RT-response time;

^a^p<.05

^b^Adjusted for processing speed

^c^Multiplied by -1 so that higher values indicate greater emotional clarity

**Table S5.** Between-person correlations between emotional clarity indices and other measures, with p-values adjusted for false discovery rate (counterpart to Table 5). None of the correlations were adjusted for baseline speed of responding, and only columns with “adjusted” had processing speed as a control variable.

|  | NA Drift Rate | NA Drift Rate, adjusted^b^ | PA Drift Rate | PA Drift Rate, adjusted^b^ | NA RT^c^ | NA RT, adjusted^b,c^ | PA RT^c^ | PA RT, adjusted^b,c^ |
| --- | --- | --- | --- | --- | --- | --- | --- | --- |
| **Subjective well-being** | | | | | | | | |
| Satisfaction with Life | .16 (*P*=.027)^a^ | .14 (*P*=.05) | 0 (*P*=.96) | -.03 (*P*=.839) | .1 (*P*=.54) | .06 (*P*=.667) | .09 (*P*=.75) | .05 (*P*=.949) |
| Neuroticism | -.14 (*P*=.037)^a^ | -.16 (*P*=.024)^a^ | -.05 (*P*=.88) | -.06 (*P*=.627) | -.01 (*P*=.866) | -.04 (*P*=.667) | .02 (*P*=.846) | 0 (*P*=.949) |
| Depression | -.27 (*P*<.001)^a^ | -.28 (*P*<.001)^a^ | -.01 (*P*=.96) | 0 (*P*=.995) | -.05 (*P*=.54) | -.04 (*P*=.667) | -.01 (*P*=.846) | 0 (*P*=.949) |
| Anxiety | -.25 (*P*<.001)^a^ | -.26 (*P*<.001)^a^ | -.08 (*P*=.88) | -.09 (*P*=.527) | -.07 (*P*=.54) | -.08 (*P*=.667) | -.03 (*P*=.846) | -.03 (*P*=.949) |
| Diabetes distress | -.25 (*P*<.001)^a^ | -.2 (*P*=.005)^a^ | .04 (*P*=.88) | .1 (*P*=.527) | -.08 (*P*=.54) | .03 (*P*=.667) | -.04 (*P*=.846) | .07 (*P*=.949) |
|  | | | | | | | | |
| DERS (total) | -.15 (*P*=.12) | -.17 (*P*=.032)^a^ | 0 (*P*=.97) | -.01 (*P*=.916) | -.01 (*P*=.91) | -.04 (*P*=.718) | 0 (*P*=.949) | -.02 (*P*=.947) |
| 1.Limited Strategies | -.12 (*P*=.184) | -.13 (*P*=.128) | .03 (*P*=.935) | .04 (*P*=.916) | -.02 (*P*=.91) | -.02 (*P*=.832) | -.01 (*P*=.949) | -.01 (*P*=.947) |
| 2.Non-acceptance | -.06 (*P*=.467) | -.09 (*P*=.239) | .06 (*P*=.674) | .04 (*P*=.916) | .04 (*P*=.91) | -.01 (*P*=.832) | .05 (*P*=.869) | 0 (*P*=.947) |
| 3.Impulse control difficulties | 0 (*P*=.992) | .03 (*P*=.666) | .09 (*P*=.674) | .12 (*P*=.499) | -.02 (*P*=.91) | .04 (*P*=.718) | -.02 (*P*=.949) | .04 (*P*=.947) |
| 4.Difficulties with goal directedness | -.07 (*P*=.467) | -.09 (*P*=.239) | -.1 (*P*=.624) | -.12 (*P*=.499) | .07 (*P*=.728) | .04 (*P*=.718) | .04 (*P*=.869) | .01 (*P*=.947) |
| 5.Lack of  Awareness | -.19 (*P*=.04)^a^ | -.21 (*P*=.014)^a^ | -.12 (*P*=.5) | -.13 (*P*=.49) | -.1 (*P*=.516) | -.14 (*P*=.168) | -.06 (*P*=.869) | -.09 (*P*=.84) |
| 6.Lack of emotional  clarity | -.14 (*P*=.144) | -.16 (*P*=.086) | -.01 (*P*=.97) | -.02 (*P*=.916) | -.07 (*P*=.762) | -.1 (*P*=.592) | -.05 (*P*=.869) | -.07 (*P*=.947) |
| Processing Speed (control variable) | .28 (*P*<.001)^a^ | - | .25 (*P*=.04)^a^ | - | .5 (*P*<.001)^a^ | - | .48 (*P*<.001)^a^ | - |

DERS- Difficulties in Emotion Regulation Scale; NA-negative affect; PA-positive affect; RT-response time;

^a^p<.05

^b^Adjusted for processing speed

^c^Multiplied by -1 so that higher values indicate greater emotional clarity
